# Chromatin Remodulator CHD4: A Potential Target for Cancer Interception

**DOI:** 10.3390/genes16020225

**Published:** 2025-02-15

**Authors:** Krishnendu Goswami, Karthikkumar Venkatachalam, Surya P. Singh, Chinthalapally V. Rao, Venkateshwar Madka

**Affiliations:** 1 Center for Cancer Prevention and Drug Development, Stephenson Cancer Center, Hem-Onc Section, Department of Medicine, University of Oklahoma HSC, Oklahoma City, OK 73104, USA; krishnendu-goswami@ouhsc.edu (K.G.); karthikkumar-venkatachalam@ouhsc.edu (K.V.); surya-singh@ouhsc.edu (S.P.S.); 2 VA Medical Center, Oklahoma City, OK 73104, USA

**Keywords:** cancer, chromatin remodulation, chromosome, CHD4, epigenetics, nucleosome

## Abstract

Cancer initiation and progression are associated with numerous somatic mutations, genomic rearrangements, and structure variants. The transformation of a normal cell into a cancer cell involves spatio-temporal changes in the regulation of different gene networks. The accessibility of these genes within the cell nucleus is manipulated via nucleosome remodeling ATPases, comprising one of the important mechanisms. Here, we reviewed studies of an ATP-dependent chromatin remodulator, chromodomain helicase DNA-binding 4 (CHD4), in cancer. Multiple domains of CHD4 are known to take part in nucleosome mobilization and histone binding. By binding with other proteins, CHD4 plays a vital role in transcriptional reprogramming and functions as a key component of Nucleosome Remodeling and Deacetylase, or NuRD, complexes. Here, we revisit data that demonstrate the role of CHD4 in cancer progression, tumor cell proliferation, DNA damage responses, and immune modulation. Conclusively, CHD4-mediated chromatin accessibility is essential for transcriptional reprogramming, which in turn is associated with tumor cell proliferation and cancer development.

## 1. Introduction

Chromatin (genetic material and its protein scaffold) is associated with compacting, protecting, and organizing DNA. It has an essential role in the preservation of genomic integrity as well as the successful transmission of genetic information over generations [1,2]. Dynamic modification of chromatin conformation regulates the availability of DNA and has an impact on gene expression. Chromatin conformation is represented by the exchange of histone variants, post-translational modification of histones and DNA, and nucleosome remodeling [3,4].

Nucleosomes are the core units of chromatin and act as a signaling hub, providing a scaffold for the positioning of chromatin remodeling factor [5,6]. One such remodeling factor, the ATP-dependent chromatin remodeling enzyme, uses ATP-derived energy to alter chromatin’s structure by changing the nucleosome position, spacing, density, or subunit composition and ultimately influencing the gene expression [7,8]. Notably, ATP-dependent chromatin remodeling enzymes are classified into four subfamilies [inositol requiring (INO80), Imitation Switch (ISWI), Swi2/Snf2-related ATPase complex (SWR1), and chromodomain helicase DNA-binding protein (CHD)] based on sequence similarity [3]. Among different chromatin remodeling factors, ISWI proteins are involved in repositioning (or sliding) nucleosomes to create regularly spaced arrays [4], SWR1 incorporates histone variants (e.g., H2A.Z), and INO80 enzymes have opposing roles in histone variant dynamics. Notably, one important chromatin remodeler family, CHD, is associated with nucleosome spacing activity and comprises subfamily I (CHD1, CHD2), subfamily II (CHD3, CHD4, CHD5), and subfamily III (CHD6, CHD7, CHD8, and CHD9) [9,10,11,12].

Within the CHD group, one key nucleosome remodeling factor is CHD4. It is a member of ATP-dependent Snf2 family helicases and a key subunit of Nucleosome Remodeling and Deacetylase (NuRD) complexes. Notably, the CHD4 gene is widely expressed in different tissues (https://www.ncbi.nlm.nih.gov/gene/1108, accessed on 11 January 2025). However, its role in cancer has not been reviewed yet. Therefore, in the current review, we primarily focused on the molecular mechanistic role of CHD4 in different cancers and its association with various cell and immune signaling. Additionally, we try to provide a brief description of CHD4’s role in DNA damage response and its role in B cell and T cell development.

## 2. Methodology

We performed a literature survey on PubMed and Google Scholar with relevant keywords like “CHD4 function” and “Chromatin remodulation factors”, and identified scholarly articles published between 1990 and 2024. From these articles containing the keywords in title or abstract, those particularly dealing with various cancers were selected. Finally, we reviewed the consequences on cancer cell development in rodent and human cancer cells with altered CHD4 function following the conditional deletion of the CHD4 gene.

## 3. CHD4 Structure and Function

**Figure 1:**
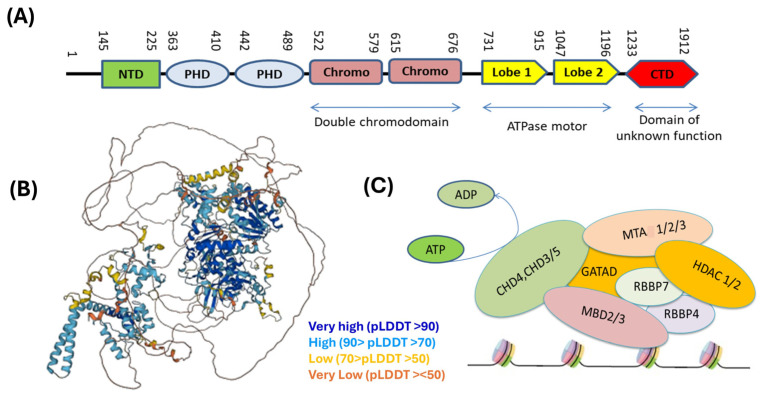
Structure of CHD4. CHD4 is composed of N terminal domain, PHD domain, Chromo domain, ATPase domain, and C-terminal domain (**A**). 3D structure of CHD4. AlphaFold per-residue model confidence score (pLDDT) indicated by different colors. [https://alphafold.ebi.ac.uk/entry/Q14839, accessed on 14 January 2025.] (**B**). NuRD complex comprises remodeler (CHD3, CHD4, or CHD5); the WD40 repeat proteins RBBP4 and RBBP7; the histone deacetylases HDAC1 and HDAC2; the metastasis-associated proteins MTA1, MTA2, and MTA3; the methyl-DNA binding domain proteins MBD2 and MBD3; and GATAD2A and GATAD2B (**C**).

The fundamental building block of chromatin is a nucleosome comprising ~146 base pairs (bp) of DNA wrapped around an octamer of histone proteins −2 (H2A, H2B, H3, and H4) [13]. Chromatin depends on ATP-dependent chromatin remodeler CHD4 for its maintenance. CHD4 is a ~200 kDa protein comprising multiple domains (Figure 1A). (a) The N-terminal domain resembles a high-mobility group (HMG) box that facilitates binding with the PAR chains of poly (ADP-ribose) and subsequently recruits CHD4-NuRD complexes at the DNA damage side [7,14,15,16,17]. (b) A pair of plant homeodomain (PHD) fingers (PHD1/2) aids CHD4 in interacting with the unmethylated or methylated N-terminal histone H3 tails on the same nucleosome [18]. (c) Two chromodomains (CD1 and CD2) involved in binding with DNA. Notably, chromodomains do not bind with histone [19,20]. (d) The ~400-residue SNF2-like ATPase/DEXH (DEAH box) helicase domain mediates histone displacement, nucleosome repositioning, and sliding by energy provided through hydrolyzing ATP and also plays a key role in CHD4-dependent transcriptional reprogramming, mostly in repression and rarely in activation of CHD4-modulating genes. Chromodomains (CD1 and CD2) can regulate the function of ATPase; additionally, (e) the C terminal portion consists of domains of unknown function (DUF) and a C-terminal domain (CTD), which has an impact on CHD4-dependent transcriptional repression in plasmacytoma [21,22,23].

CHD4 (Figure 1B), by itself or as a part of the NuRD complex, plays a vital role in chromatin remodulation and in the regulation of gene expression. Recently, high-resolution analysis of the interaction between the CHD4-ATPase motor and nucleosomal DNA showed that electrostatic interactions occurred between lysine and arginine residues of CHD4 and the phosphate backbone of the tracking and guide strands. It was also shown that CHD4 binds with the histone H4 tail through its ATPase lobe 2 [13,24]. Acetylation of H4 Lys16 weakens these charge-based interactions, resulting in a reduction in affinity between CHD4 and the H4 tail, followed by the relaxation of transcriptionally active regions. On the other hand, the CHD4 double-chromodomain region can interact with the methylated Lys27 of the H3 tail, resulting in gene repression. Additionally, CHD4-ATPase lobe 2 residues [Asn1004 and Leu1009] can interact with histone H3 (α helix 1, Gln76, and Arg83) [24]. Recent reports identified that CHD4 is recruited via transcription factor GATA4 (GATA binding protein 4), NKX2-5 (NK2 homeobox 5), and TBX5 (T-box transcription factor 5), and it promotes the regulation of cardiac genes [25].

A mutagenesis-mediated study confirmed the role of CHD4 in the NuRD complex [26]. The NuRD complex (Figure 1C) comprises histone deacetylases (HDAC 1) and (HDAC 2) [these are responsible for deacetylating lysine residues in histone tails] [27], methyl-binding domains (MBD2 or MBD3) [role in stabilization of the methylation of inaccessible DNA] [28,29], metastasis-associated (MTA 1, 2, or 3) proteins, [mediate interactions between NuRD and sequence-specific DNA-binding proteins] [30], GATAD2A and GATAD2B [zinc finger proteins that aid in the recruitment of CHD4 to NuRD complexes] [31], RBBP4/7 (WD40 repeat-containing Retinoblastoma-binding proteins 4 and 7, modulates histone deacetylase activities of NuRD complexes) [32,33].

**Figure 2:**
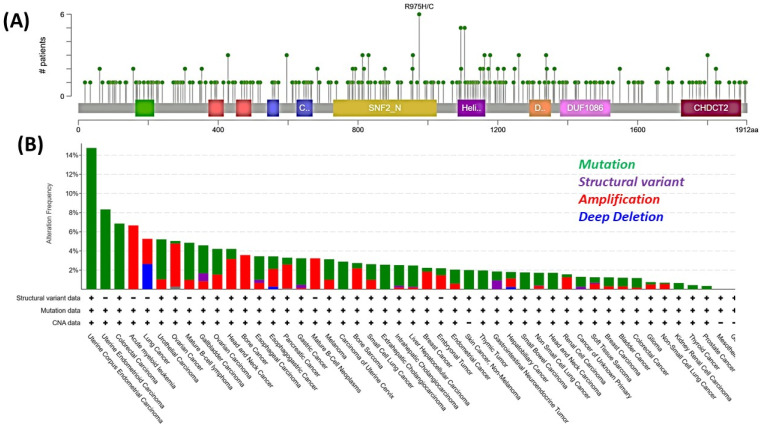
CHD4 mutations and their frequency in various cancers. Tumor mutation data analysis suggests ~278 missense mutations in the CHD4 gene. e.g., R975H/C (Arg975His or Arg975Cys) found in 6 patient tumors. (**A**). Frequency of various genetic alterations (+/− indicate presence/absence respectively) in CHD4 genes across various tumor types (**B**). (Data source: cBioPortal, accessed on 14 January 2025.).

Genetic alterations in CHD4 are frequently observed in various cancers. Tumor mutational data suggest frequent occurrence of multiple missense mutations in CHD4, which span across all regions of this gene (Figure 2A). Additionally, structural variants like deletions, amplifications, and fusions of CHD4 are found in different tumor types (Figure 2B). Interestingly, CHD4 missense mutations [Cys464Tyr, Val558Phe, Arg572Gln, Leu912Val, His1151Arg, Arg1162Gln, His1196Tyr, and Leu1215] are found in endometrial cancers and several other diseases like Sifrim–Hitz–Weiss syndrome (γ) [Cys467Tyr, Ser851Tyr, Gly1003Asp, Arg1068His, Arg1127Gln, Trp1148Leu, Arg1173Leu, and Val1608Ile] [34,35,36]. Other than this, there are several mutations associated with various kinds of cancer and syndromes. Notably, Sifrim–HitzWeiss syndrome is an autosomal-dominant intellectual developmental disorder that exhibits a de novo pathogenic CHD4 variant. This syndrome is associated with intellectual disability, distinctive facial dysmorphism, developmental and speech delay, macro/microcephaly, congenital heart defects, hearing impairment, etc.

CHD4 missense mutation is also found in left ventricular noncompaction cardiomyopathy (LVNC) and is a complex myocardial disorder [associated with abnormal development of the ventricular chambers] characterized by endangered cardiac dysfunction and a high risk of sudden death [25]. All this evidence suggests that CHD4 plays a vital role in genome integrity, being compromised in tumors and other health conditions.

## 4. Cell Death Response and CHD4

Cells sustaining DNA damage are a hallmark associated with different human cancers. Cells adapt to DNA damage involves increased reactive oxygen species (ROS) and several cascade reactions to initiate the DNA damage repair (DDR) pathway that favors the survival of premalignant cells and their eventual transformation into malignant cells. DDR promotes epigenetic alteration (changes in nucleosome positioning and histone modifications) in surrounding chromatin, which facilitates the recruitment of repair factors at the DNA break region and consequently holds the transcription process [37,38,39,40,41,42,43,44].

**Figure 3:**
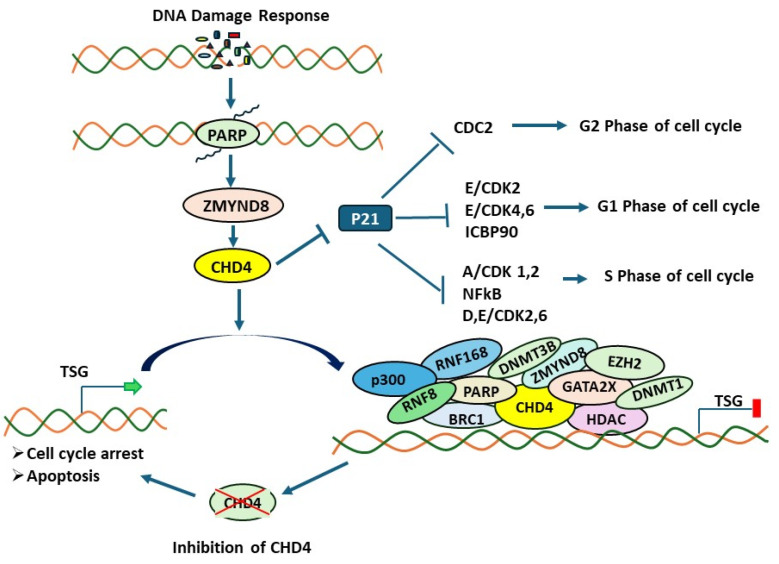
Disrupting CHD4 leads to cell cycle arrest and apoptosis. On the DSB side, PARP1 recruits CHD4 in a ZMYND8-dependent way. Both CHD4 and ZMYND8 facilitate the recruitment of other signaling molecules and the NuRD complex, followed by suppressing the transcription of tumor suppressor genes (TSGs).

During DDR in the normal cell, PARP1 recruits CHD4 at the double-stranded break (DSB) side in a ZMYND8 (zinc finger and MYND [myeloid, Nervy, and DEAF-1] domain containing 8)-dependent way (Figure 3).

It was shown that the MYND domain of ZMYND8 facilitates the recruitment of the GATAD2A/NuRD complex on the same side, possibly in a PARP (poly (ADP-ribose) polymerase)-dependent manner [45,46]. Another report suggests that, mechanistically, the bromodomain (the primary “readers” of acetylated chromatin) of ZMYND8 recognizes TIP60-acetylated histone H4 (TIP60; histone acetyltransferase (HAT) cofactor acetylates H4K16, as well as H2AK15 during damage response) and recruits NuRD on the DSB side to suppress transcription and eventually support the homologous repair (HR) pathway. In concordance with the DSB chromatin context, HAT p300, RNF8/RNF168, and BRCA1 are also involved in CHD4 recruitment in a PARP1-dependent manner [42,43].

In HR-deficient cells, the absence of CHD4 causes disruptions in the assembly of RAD51, RPA, and BRCA2 on the side of the double-stranded break (DSB), resulting in cells being hypersensitive to the PARP inhibitor. Moreover, at DSBs, CHD4 facilitates the recruitment of HDAC1 and HDAC2, resulting in the hypoacetylation of H3K56 followed by the manipulation of NHEJ factors such as Ku70 and Artemis [22,42]. Infra-red induced double-strand breaks activate PARP, which incorporates poly ADP ribose (PAR) chains at sites of DNA damage [45]. PAR chains recruit different DNA repair proteins and the NuRD complex. CHD4 was found to contain PAR-binding motifs in its amino-terminal region. Depletion of CHD4 results in hypersensitivity to DNA damage and the accumulation of unrepaired breaks at the sites of DNA damage [45]. Loss of CHD4 also results in CDC25A degradation and p21 accumulation, leading to cell cycle delay [34,43,45]. It is now clear that CHD4 expression is upregulated in various cancers. So, it is important that inhibiting CHD4 in cancer cells makes them more sensitive to DNA damage and consequently promote cancer cell death.

Notably, the molecular association between the DNA damage checkpoint protein ATR and ATM is found to be connected with CHD4 recruitment to the damaged sites. It is also reported that CHD4 is linked to the regulation of DNA damage responses and cell cycle progression. It is possible that CHD4 plays synergistic and/or antagonistic functions in cell signaling and its function to timely repair damaged DNA and maintain cellular viability. However, the recent study results cannot explain the entire complexity of CHD4’s contribution to DNA damage response and cell viability [45,47,48].

CHD4 influences chromatin remodeling, and chromatin remodeling affects many processes, including DNA repair, replication, and transcription; therefore, CHD4’s roles could clearly be quite complex. Though the most prominent function of CHD4 is to act like an oncogene, during the DNA repair process, it aids in preventing cell growth just to finish the cell repair process [45].

## 5. Importance of CHD4’s Role in Immune Cell Development and the Signaling Cascade

B cell and T cell development are recognized as a well-studied paradigm of lineage specification and critical events in shaping the immune system [49].

### 5.1. CHD4’s Role in T Cell Development

Regarding T cells, immature thymocyte CD4^+^CD8^+^ generation starts from committed lymphoid progenitors in the bone marrow, from which they pass through the blood and are destined to the thymus. Here, these cells are designated as committed T cell precursors, also known as double-negatives (DN; no CD4 or CD8), and lose their potential to form other immune cell types like natural killer (NK) cells and B cells. DN cells simultaneously progress to the double-positive (DP) stage [49]. During the DP to single-positive (SP) transition, the T cell repertoire is formed by a process known as positive selection [49,50].

**Figure 4:**
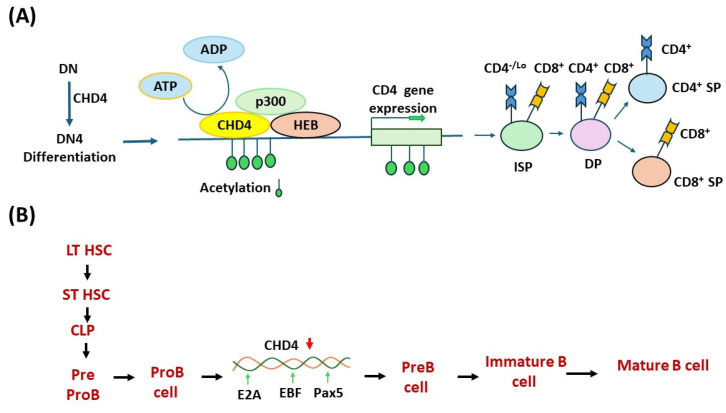
CHD4 promotes the differentiation of double-negative (DN; no CD4 or CD8) T cells and also promotes B cell maturation. CHD4 and p300 interact with HEB on the CD4 enhancer. The presence of p300 and CHD4 complex induces local histone hyperacetylation (flags), followed by open configuration of chromatin, and finally induces CD4 gene expression (**A**). In pro-B cell to B cell lineage, commitment is achieved by the expression of Paired box 5 (PAX5) and EBF1 negatively regulated by CHD4, followed by activation of Cd79 genes that are required for the synthesis of Ig-α and Ig-β chains of the B cell receptor (BCR) (**B**).

Based on the findings of the Georgopoulos group, CHD4 works in association with HAT p300 and E box proteins, and HEB is recruited at the CD4 enhancer and aids in increasing local histone acetylation, followed by the formation of an open configuration of chromatin and then followed by activation of CD4 expression (Figure 4A) [51,52]. Notably, CHD4 is crucial to the recombination of TCRα and TCRβ genes in T cells, and a loss of CHD4 causes a decrease in DP and CD4+ but not CD8+ thymocytes. CHD4 forms a complex with GATA3 [GATA-binding protein family] and p300 to positively regulate Th2-type cytokine genes. Although interacting with HDAC2, CHD4 downregulates the TBX21 gene and also inhibits Th1 differentiation and interferon-γ expression [53].

### 5.2. Importance of CHD4’s Role in Early B Cell Development

B lymphocytes produce immunoglobulins (Igs), which are made of two heavy (Igh) and two light (Igκ or Igλ) chains. The N terminus variable regions exons of Igh and Igκ or Igλ are assembled via V(D)J recombination in pro- and pre-B cells within the bone marrow. Thereafter, the naive IgM^+^ IgD^+^ B cells pass through secondary lymphoid organs (spleen, lymph nodes, Peyer’s patches). In secondary lymphoid organs, they encounter antigens and undergo Igh class switch recombination (CSR) and somatic hypermutation (SHM). SHM occurs in the center (GC) of lymphoid follicles, where the N terminal variable regions of Igh and Igκ or Igλ are mutated 10^−2^ to 10^−3^/bp/generation and higher-antigen-affinity B cells are selected. CSR happens within GCs or in the extra-follicular regions, where the Cμ constant region exchanges with gene segments (Cγ, Cε, Cα) so that B cell expressing IgM changes to IgG, IgE, or IgA. Interestingly, CHD4 promotes CSR and aids in the proliferation of B cells [54].

Moreover, the conventional B cell development process comprises stage-specific gene expression and the reprogramming of immunoglobulin gene arrangements [7]. B cell development is initiated from long-term hematopoietic stem cells, and then intermediate stages (e.g., lymphoid-primed multipotent progenitors), and next common lymphoid progenitors, which can give rise to B-, T-, and NK-cells. In pro-B cells, lineage commitment is achieved by the expression of Paired box 5 (PAX5) and Early B cell Factor 1 (EBF1), which transcriptionally activate the expression of CD79a and CD79b genes that are involved in the synthesis of the Ig-α and Ig-β chains of the B cell receptor (BCR) and parallelly restrict the synthesis of myeloid differentiation marker CD244 [55,56,57,58,59,60,61,62,63].

Abolition of CHD4 disrupts the IL-7 mediated signaling cascade and reduces the level of the nuclear mediators of IL-7 signaling, i.e., pSTAT5a and pAKT. Notably, IL-7 drives B cell proliferation and is responsible for the transformation of pro-B to pre-B cells [64,65]. Therefore, the role of CHD4 in early B cell development is undeniable (Figure 4B). However, it is still unknown how CHD4 plays a regulating role in B cell and T cell development during cancer progression.

## 6. Association of CHD4 with Molecular Cell Signaling Pathway in Various Cancers

**Figure 5:**
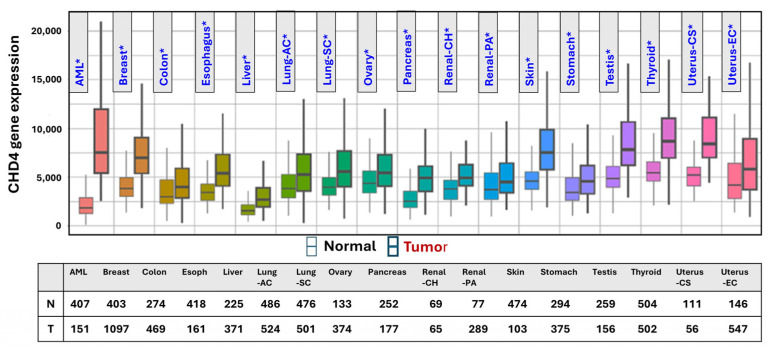
CHD4 is significantly overexpressed in several cancers (* Mann–Whitney *p* < 0.05). TCGA data were analyzed using the TNMplot web tool [66].

Here, we show an analysis of Cancer Genome Atlas (TCGA) data using TNMplot [66], which suggests that CHD4 expression is significantly upregulated in different cancer tissues compared to corresponding normal tissue levels (Figure 5).

### 6.1. CHD4 Is Required for Promoting Childhood Acute Myeloid Leukemia (AML)

CHD4 expression is significantly high in AML (Figure 5) and is reported to relate to the growth of leukemic cells [67]. In AML development, CHD4 is associated with an impaired differentiation of hematopoietic progenitors, resulting in increasing immature myeloid blast cells in the bone marrow. The abolition of CHD4 by a shRNA vector in two different human AML cells, THP-1 and NOMO-1, showed a suppression of MYC and its target genes, followed by cell cycle arrest in the G0 phase, which suggests the importance of CHD4 for cell growth of leukemic cells and disease progression. It was also identified that CHD4 has no role in the cell growth of normal hematopoietic cells [67,68]. Additionally, the abolition of CHD4 promotes AML blasts that are more sensitive to genotoxic agents daunorubicin (DNR) and cytarabine (ara-C) [69].

### 6.2. CHD4 and Breast Cancer

Breast cancer, a heterogeneous genetic disease, is one of the leading causes of death among women worldwide. It comprises four clinical subtypes: estrogen receptor-positive (ER^+^), progesterone receptor-positive (PR^+^), and human epidermal growth factor receptor-positive (HER2^+^) forms, and lack of expression any of the above is known as triple-negative breast cancer (TNBC). In the field of oncology, TNBC remains an alarming challenge due to its molecular heterogeneity and involvement of several signaling cascades like PI3K/AKT/mTOR, MAPK/ERK, and JAK/STAT, followed by limited treatment options [70,71,72].

In TNBC, CHD4 regulates transcription of b1 integrin, E-cadherin, and p21, resulting in the modulation of cell migration proliferation and invasion [73]. A recent report showed that F-box and WD repeat domain-containing 7 (FBXW7), an E3 ligase-degraded CHD4 protein in TNBC cells, resulted in a decrease in stemness properties in TNBC cells as well as affecting the Wnt/β-Catenin pathway [74]. The abolition of CHD4 in SKBR-3 and BT474 breast cancer cells intervened in the ERBB2 signaling cascade. ERBB2 is an oncogene connected with the Ras/Raf/MAPK and PI3K/AKT pathways and it enhances the expression of ATG12 (autophagy-related 12), consequently activating autophagy and developing resistance against several drugs. Depletion of CHD4 is shown to upregulate p62 and the ratio of LC3II/LC3I (autophagy marker), resulting in late autophagy in SKBR-3 and BT474 cells [75]. It is also reported that the ablation of CHD4 causes a downregulation of PI3K protein levels, as well as decreased phosphorylation of AKT and ERK (important pro-survival and proliferation kinases), and induces p27KIP1 upregulation, simultaneously inhibiting ERBB2^+^ breast cancer cell proliferation [76]. Breast cancer initiation and progression are connected by estrogen receptor α (ERα), positively coregulated with CHD4 expression. In T47D and MCF-7 cells, knockdown of CHD4 caused decreased ERα expression, and its overexpression elevated ERα at the CCND1 promoter, respectively [77]. Loss of CHD4 is reported to enhance sensitivity to the DNA-damaging agent cisplatin. Depletion of CHD4 in the BRCA1 mutant HCC1937 breast cancer cell line enhances the formation of g-H2AX foci, followed by a reduction in growth via cisplatin [73,74,75,76,77].

Abolition of CHD4 impairs the recruitment of HDAC1 to the p21 promoter, resulting in the upregulation of anti-proliferative effector P21 (a cyclin-dependent kinase inhibitor protein), suggesting that CHD4 is a potential therapeutic target in breast cancer [78]. In TNBC cells, malignant behaviors correlated with a high expression of CHD4, which regulates the activity of E-cadherin, N-cadherin, and fibronectin and plays a role in epithelial–mesenchymal transition (EMT) [68]. β1 integrin is essential for cancer cells and for mediating as an adhesion molecule between cancer cells and the extracellular matrix (ECM). CHD4 has been shown to regulate β1 integrin in TNBC and has been reported to enhance its expression in TNBC patients. SETDB1 is a histone methyltransferase that helps in the binding of methyl radicals to lysine9 of histone H3 (H3K9me2 and H3K9me3); later, CHD4 can recognize H3K9me3 marks in TNBC cells Hs578T [78,79,80,81,82].

It is also found that CHD4 regulates SRY-box 2 (SOX2: regulator of stem cell pluripotency and has a role in the function of cancer stem cells, CSCs) transcription through TRPS1 (GATA-type transcription factor that promotes angiogenesis) in luminal breast cancer. The TRPS1-CHD4/NuRD (MTA2) complex regulates TP63 expression [83,84,85,86]. In the normoxia conditions of breast cancer cells, CHD4 is recruited at HIF (Hypoxia-Inducible factor) target gene promoters, binds with (H3K9ac), and facilitates enhanced RNA polymerase II loading through p300, which is followed by the activation of HIF target genes ANGPTL4, NDNF, LOX, and VEGFA; the same result was shown in human breast tumors, suggesting HIF transactivation by CHD4 [17]. Overall, CHD4 is associated with a complex signaling cascade and plays a vital role in the progression of breast cancer, and CHD4 might be associated with the worse prognosis of TNBC.

### 6.3. CHD4 in Colorectal Cancer (CRC)

CRC constitutes about 15 percent of all cancers worldwide and is among the top three cancers in men and women. Both genetic and epigenetic alterations promote the development of a benign adenoma to a carcinoma or metastatic disease. Analysis of the transcriptome data of tumors showed that the epigenetic modulator CHD4 is overexpressed in CRC (Figure 5) and correlates with poor survival [1,38]. Knockdown of CHD4 in DLD-1 CRC cells reduced vimentin, MMP2, and N-cadherin, whereas E-cadherin was increased, suggesting that CHD4 regulates the migration and invasive abilities of DLD-1 cells. CHD4 is suggested to play an oncogenic role by modulating the chromatin accessibility of the promoter region by promoting DNA hypermethylation as well as repressing the transcription of several CRC-relevant tumor suppressor genes, like E-cadherin (CDH1), WNT inhibitory factor 1 (WIF1), TIMP metallopeptidase inhibitor 2 (TIMP2), TIMP metallopeptidase inhibitor 3 (TIMP3), mutL homolog 1 (MLH1), cyclin-dependent kinase inhibitor 2A (CDKN2A), secreted frizzled-related protein 4 (SFRP4), and secreted frizzled-related protein 5 (SFRP5) [1,38].

Rectal cancer represents 30% of CRCs, and its incidence has been progressively increasing. Analysis of the Gene Expression Omnibus (NCBI-GEO) database-derived microarray datasets (GSE68204) of rectal cancer demonstrated a significant upregulation of CHD4 expression in rectal cancer patients. Histological studies, combined with transcriptomic data and protein–protein interaction studies, showed that CHD4 acts as a potential biomarker in the context of rectal cancer. Analysis of the DNA repair machinery related to rectal cancer patients revealed high rates of mutations occurring in ATM and MRE11, and these were mostly correlated with PARP (poly-ADP ribose polymerase) pathway-mediated homologous recombination pathway-related genes. CHD4 is known to regulate the DNA damage response in a PARP-dependent manner [10,43]. A recent report suggested that the depletion of CHD4 sensitizes cancer cells to (PARP) inhibitors in hematopoietic tumors. It is concluded that targeting CHD4 will facilitate sensitivity towards PARP inhibitors in rectal cancer cells [87].

### 6.4. CHD4 and Stomach/Gastric Cancer (GC)

GC represents one of the predominant cancers, involving a complex degree of malignancy, strong invasiveness, and heterogeneity in the context of the alteration of genetic regulation. Significant CHD4 overexpression is observed in stomach cancer (Figure 5). One of the leading genetic regulation pathways that comprised Circular RNA (circRNA) [endogenous noncoding RNAs with covalently closed loops] mediated genomic reprogramming and was demonstrated in several cancers [88]. Recently, the involvement of the hsa_circ_0007396-miR-767-3p-CHD4 axis was shown in the progression and carcinogenesis of GC [83]. Notably, circRNA can interact with transcription factors and can modulate transcription as well as circRNA-mediated miRNA sponge function, where miRNA, upon binding with the untranslated region, regulates the translation of mRNA of the target genes. There are limited studies on the role of CHD4 in GC. Thus, detailed studies on CHD4 in GC may be helpful in this disease’s management.

### 6.5. CHD4’s Role in Glioblastoma Multiforme (GBM)

RAD51 (a protein scaffold associated with homologous recombination machinery) expression is elevated in malignant solid tumors. CHD4 directly binds to the RAD51 promoter at (H3K9Ac) and regulates its expression, and it produces permissive and active chromatin in GBM cell lines. CHD4 depletion increased γH2Ax (an anti-oncogenic barrier) levels, enhanced DNA damage, and decreased RAD51 expression in GBM cells. Moreover, CHD4 drives RAD51 expression and is responsible for resistance to radiation in GBM cells [89].

### 6.6. Liver Cancer and CHD4

Liver cancer, or hepatocellular carcinoma (HCC), is one of the dominant cancers worldwide, associated with various alterations in genomic architecture. Recent evidence suggests that a subset of cells, cancer stem cells (CSCs), play a key role in tumor maintenance. CHD4 is involved in the formation of several stem cell markers, including epithelial cell adhesion molecule (EpCAM), and can be used to classify HCC subtypes [90]. EpCAM^+^ CSCs activate elevated levels of a transcription factor, Sal-like protein 4 (SALL4), a biomarker of HCCs [91]. SALL4 has been shown to bind with the NuRD complex [92]. Recently, it has been reported that EpCAM^+^ liver CSCs can be intervened with by targeting the NuRD component of CHD4 and the knockdown of CHD4, thus affecting HCC cell migration and invasion. It is also demonstrated that the CHD4/NuRD complex facilitates the impairment of CD8^+^ T cell and DC cell infiltration in hepatocellular carcinoma [93].

### 6.7. CHD4—An Important Molecular Signature for Malignancy in Lung Cancer

Small cell lung carcinoma (SCLC) and non-small cell lung cancer (NSCLC) are common types of lung cancer associated with highly aggressive clinical features and poorer prognosis [94]. In an exome-chip study, nine missense single nucleotide variations (SNVs) were found in CHD4, where rs7479004 (p.D140E) SNVs were more significant and showed a link with individuals who smoke [95]. CHD4 mutation rs74790047 was identified as a common exonic mutation connected with lung cancer patients [96,97]. Interaction between CHD4 and PHF5A (PHD finger protein 5A) and the Rho/(Rho-associated protein kinase) ROCK signaling plays a key role in NSCLC cell growth, migration, and invasion. Molecular studies have revealed that CHD4 binds with PHF5A to regulate Rho/ROCK signaling in NSCLC cells. Moreover, CHD4 was also found to regulate extracellular signal-related kinases, which play a dual role (oncogenic/tumor suppressor gene) in cancer [98].

### 6.8. CHD4—Promotes Papillary Thyroid Cancer (PTC)

Notably, the CHD4 role is also found in papillary thyroid cancer (PTC). PTC is the common subtype of thyroid carcinoma and is increasing worldwide. CHD4 promotes cancer stemness and EMT in PTC cells [99]. Knockdown of CHD4 in PTC cell lines BCPAP and TPC-1 decreased the expression of N-cadherin, vimentin, Twist, Snail1, Zeb1, MMP-2, and MMP-9 and increased E-cadherin expression, followed by decreased invasion and migration of these cells [100].

### 6.9. Potential Role of CHD4 in Ovarian Cancer

Ovarian cancer is the deadliest gynecologic-related malignancy, where the most common subtype is epithelial ovarian cancer (EOC). The silencing of CHD4 expression in malignant ovarian cancer cell lines (SKOV3 and OVCAR3) results in reduced cell viability as well as decreased aggressive behaviors of the cell. Recent studies suggest that by modulating the HDAC function, CHD4 alters cellular histone status, alters the PARP-mediated DNA damage response, and sequentially promotes the progression of ovarian cancer [101]. Notably, CHD4 can recruit DNMT1, DNMT3B, G9a, and EZH2 [101]. Surprisingly, higher expressions of DNMT3B and EZH2 are reported in ovarian cancer and, as per RNA-seq results, CHD4 expression is positively correlated with EZH2 in ovarian cancer patients. CHD4 was found to cooperate with EZH2 and promote the accumulation of β-catenin in the nucleus by enhancing the Wnt/β-catenin pathway [96]. In HCC, EZH2-mediated methylation at the K49 site of β-catenin protein prevented the ubiquitination-mediated degradation of β-catenin, followed by nuclear accumulation as already reported [96]. Whether the above mechanism is present in EOC needs to be further explored. Overall, CHD4 plays a vital role in the maintenance of ovarian cancer in vitro and in vivo [102]. Knockdown of CHD4 reduces the expression of multi-drug efflux transporter ATP-binding cassette ABCB1 or multi-drug resistance-1 (MDR1) in ovarian cancer cell lines (TOV21G and JHOG5); this suggests that CHD4 modulates the function of MDR1 and plays a vital role in ovarian cancer [102,103]. In summary, the above observations highlight CHD4’s vital role in the maintenance of ovarian cancer and its potential as a promising target to prevent this disease.

### 6.10. Tumor Suppressor Role of CHD4 in Uterine Cancer

Uterine (endometrial) cancer is the most common cancer of the female reproductive system, associated with various mutations. TGF β signaling promotes uterine cancer stemness and is known to be regulated by CHD4 mutation [104]. A recent report suggested that knockdown of CHD4 or its mutations (R975H or R1162W) is implemented with increased expressions of TGFB1 and CD133 (cancer stem cell marker), followed by the progression of uterine (endometrial) cancer. The TGFB1/CD133 pathway is reported to be involved in promoting cell invasion, inducing stemness characteristics, and playing a vital role in drug resistance. Additionally, TGFB1 relates to a reduction in DNA double-strand break repair, thus enhancing genomic instability. Elevated expressions of TGFB1 and CD133 were reported in the activation of endometrial cancer cells. Therefore, unlike in other cancers, CHD4 acts as a tumor suppressor gene in endometrial cancer via modulation of the TGFB1/CD133 pathway [104]. To understand the molecular mechanism of CHD4 in different cancers, we summarize the roles of CHD4 in Table 1 below.

## 7. Targeting CHD4 for Cancer Therapy

CHD4 is multifunctional and takes part in several aspects of chromosomal biology, being connected to transcriptional activation or suppression, DNA repair, and DNA replication. The details of the molecular function and specificity of CHD4 in different cancers discussed above highlight the essential role of CHD4 in the maintenance of these cancers and might set a path for designing effective cancer therapeutic agents.

Notably, emerging evidence points out that an overexpression of CHD4 and CHD4-mediated suppression of tumor suppression genes is more prominent in several cancers. There is a lack of clarity on whether CHD4 is utilized by specific transcription factors in diverse biological contexts of cancer progression, compelling the need for additional biochemistry and structural biology-related study on CHD4 to identify selective targets for therapeutic purposes.

It is possible that the microenvironment and unique transcriptional program of each cell type play a vital role in deciding the CHD4 interactome partner, as well as the CHD4- mediated chromatin signaling cascade. Understanding of the molecular mechanisms underlying CHD4’s dual role in transcriptional activation and repression and the signals that trigger these roles in both normal and abnormal tissue needs to be investigated to facilitate the generation of a model connected to the molecular function of CHD4 in cancer [105,106].

In the context of CHD4-targeting drugs, recently, Ch41, a novel inhibitor of CHD4, was shown to inhibit the growth of lung tumors and hepatocellular carcinomas in immunocompetent C57BL/6 mice, but it was impaired in immunodeficient C57BL/6 mice [107]. Another CHD4 targeting inhibitor (ED2-AD101) has been studied for AML treatment [108]. Targeting CHD4 might be highly promising, and the screening of more CHD4 inhibitors and mutating of specific targeted sites needs to be further explored through in- vivo and in vitro models that can predict the regulation mechanism, resulting in a promising agent in cancer biology. Notably, CHD4 has ATPase catalytic subunits, so using competitive ATP inhibitors and allosteric agents or targeting CHD4 binding protein–protein interaction interfaces might be possible approaches, which requires further investigation.

## Figures and Tables

**Table 1:** Biological and clinical significance of CHD4 in different cancers.

Cancer Types	Biological Significance and Downstream Signaling of CHD4	Clinical Significance of CHD4	References
Acute myeloid leukemia (AML)	CHD4 positively regulates MYC and its target genes	Overexpression of CHD4 promotes significant growth of leukemic cells.	[67,68,69]
Breast cancer	CHD4 regulates transcription of b1 integrin, E-cadherin, and p21. Intervenes in ERBB2 signaling cascade	Increase stemness properties in TNBC. Modulates cell migration, proliferation, and invasion.	[70,71,72,73,74,75,76,77,78,79,80,81,82,83,84,85,86]
Colorectal cancer (CRC)	Enhances vimentin, MMP2, and N-cadherin expression	Positively regulates the migration and invasive abilities of CRC cells.	[1,38,43,87]
Stomach/gastric cancer (GC)	Alteration of genetic regulation via CHD4-associated Circular RNA (circRNA)	Significant overexpression of CHD4 is observed in stomach cancer.	[88]
Glioblastoma multiforme (GBM)	CHD4 binds to the RAD51 promoter at (H3K9Ac) and regulates its expression	Promotes malignant solid tumors.	[89]
Liver cancer	CHD4 is involved in the formation of stem cell marker [epithelial cell adhesion molecule (EpCAM)]	Promotes HCC cell migration and invasion.	[90,91,92,93]
Lung cancer	CHD4 mutation rs74790047 was identified as a common exonic mutation in lung tumors	Elevates NSCLC cell growth, migration, and invasion.	[94,95,96,97,98]
Papillary thyroid cancer (PTC)	Regulates expression of N-cadherin, vimentin, Twist, Snail1, Zeb1, MMP-2, and MMP-9	CHD4 promotes cancer stemness and EMT in PTC cells.	[99,100]
Ovarian cancer	CHD4 alters cellular histone status and alters the PARP-mediated DNA damage response	Responsible for aggressive behaviors of epithelial ovarian cancer (EOC) cells.	[101,102,103]
Uterine cancer	CHD4 regulates TGF β signaling	Act as a tumor suppressing gene.	[104]

## Data Availability

No new data were created or analyzed in this study.
